# Preserving Ecosystem Services in Urban Regions: Challenges for Planning and Best Practice Examples from Switzerland

**DOI:** 10.1002/ieam.1392

**Published:** 2013-01-10

**Authors:** Silvia Tobias

**Affiliations:** Swiss Federal Institute of Forest, Snow and Landscape Research WSL, Landscape Ecology GroupZürcherstrasse 111, CH-8903 Birmensdorf, Switzerland

**Keywords:** Urban fringe, Land use planning, Soil sealing, Ecosystem services, Best-practice examples

## Abstract

This article presents a literature review that explores the challenges for planning in urban regions in connection with the preservation of ecosystem services. It further presents some best practice examples for meeting these challenges. The demand for the provision of ecosystem services within urban regions changed during the transition from a largely agrarian society to an industrial society and, most recently, to a service society. Although in the past, provisioning services such as food production or the provision of raw material were decisive for urban development, today cultural services, e.g., clear views or nearby recreation areas, have become increasingly important. According to the literature, soil sealing is the greatest threat urbanization poses toward ecosystem services, as it compromises all of them. Spatially extensive cities with a high building density particularly inhibit regulating services like the regulation of temperature or water surface runoff. Conversely, scattered settlement patterns may lead to very small remnants of open space that cannot reasonably serve as natural habitat, agricultural land, or recreation area. The challenges for planning in urban regions are: 1) specifying regulations that define outer limits to the development of each settlement unit, 2) comprehensive planning with focal points for development, and limiting access and development at other places, and 3) compensating for new soil sealing by restoring nearby sealed areas. The article presents 3 best-practice examples that support these principles: designating areas with a particular soil quality that should not be built over, offering incentives for corporate planning in urban regions, and restoring a country road in connection with a motorway construction. Integr Environ Assess Manag 2013; 9: 243–251. © 2013 SETAC

## INTRODUCTION

Preserving ecosystem services is a crucial task in urban regions. On the one hand, there is a particularly strong consumption of ecosystem goods and services because much of the human population is concentrated in cities and their environs. On the other hand, land take for settlements and the consequent soil sealing have a disastrous impact on many ecosystem services. Over the past 3 decades, urban regions have grown stronger than ever before in terms of both population and surface. Today, almost 80% of all Europeans live in cities or their outskirts. The spatial extent of urban areas, however, has grown almost 4 times stronger than their population (EEA 2006; Prokop et al. 2011). Not only has the area of sealed soil dramatically expanded, but the urban settlement patterns have also become increasingly dispersed (Hammer et al. 2004; Kasanko et al. 2006).

Spatial planning has a strong impact on the ecosystem services, particularly in urban regions, because it is the policy domain to steer the development of residential areas and infrastructure. However, the current planning instruments are not geared to preserve ecosystem services. So the question arises: how can spatial planning contribute to the preservation of ecosystem services in urban regions? Answering this question requires knowledge about the relationship between urbanization and ecosystem services, the challenges for planning and approaches to meet them. The basic question above has been split into 4 subquestions giving the structure of the article:

1. What are the connections between ecosystem services and urban development?
2. What are the effects of urbanization on ecosystem services?
3. What are the challenges for spatial planning in urban regions?
4. What are suitable approaches in the practice of planning and civil engineering to mitigate the negative effects on the ecosystem services particularly important in urban areas?

The article closes with a short discussion and conclusion.

## METHODS AND MATERIALS

I conducted a literature review on 2 subjects: drivers and processes of urban development as well as ecosystem services in urban regions. I first started with general key words, then I used more specific key words that had been given in the articles found, and I finally looked up articles that had been cited in the literature found. This procedure enabled a targeted search with the focus on the research questions mentioned in the Introduction. The media I used were the Thomson Reuters (ISI) Web of Science, the catalogue of Libraries and Information Centers in Switzerland (NEBIS), and the internet search engine, Google. The latter was particularly useful in finding reports from authorities. The main focus of the article is on urban regions in industrialized countries, particularly in Europe and North America. Hence, I only encompassed articles and books reporting on these regions, and excluded, for example, literature about megacities in developing countries.

The best practice examples from Switzerland (see below) were selected using the following criteria:

1. They represent existing instruments that have been applied in planning and engineering practice and that practitioners are experienced with
2. They show innovative approaches in planning and civil engineering
3. They are suited to handle the challenges this article identifies for planning in urban regions
4. The author was involved in the development or evaluation of these examples considering ecosystem services

## DRIVERS OF URBAN DEVELOPMENT

In the past, the drivers of town development were location-specific production, trade, and the establishment of market places, as Cronon (1991) discussed for the city of Chicago. Natural advantages like the occurrence of nearby raw material, fertile soils, or favorable climatic conditions determined the production, which was bound to specific geographic situations. Consequently, the most productive agricultural soils are located close to urban areas (Nizeyimana et al. 2001). The presence of transport facilities like canals, roads, and railways also promoted town development (Berliant and Konishi 2000). Decisive economic factors affecting urban development were, and still are, the distances between consumers and market places as well as the capacity of the mass transportation systems. Before the age of the railways, waterways were the traditional transport routes for goods. Consequently, sophisticated canal systems were established in many European countries in the 18th and 19th century and have persisted until today. The subsequently intensified railway and road networks accelerated the expansion of the settlements. The German geographer, Walter Christaller (1980), observed the development of hierarchical town systems, where certain cities render services, from which not only the local population benefits but also the inhabitants of neighboring towns. He explained this phenomenon in 1933 with his theory of central places (Christaller 1980). Fujita et al. (1999) modeled the development of hierarchical town systems with the parameters of critical population sizes and the distances between the producers of industrial goods in the cities and the consumers of those products in the agricultural hinterlands. Their model outlines the necessary expansion of the agricultural area in far away regions due to population increase, and explains the origin of new cities as additional locations of industrial production. The model describes the evolution of the urban system in the United States in the 19th century very well.

Today, the tertiary sector predominates the economic activities in cities and is independent of specific natural factors associated with the location. The location of economic centers depends mainly on the availability of qualified labor, i.e., knowledge stocks, and the access flexibility of traffic and communication networks (Kobayashi and Okomura 1997). Traffic network planning influences the economic development of a region considerably. The ideal railway connection of the satellite towns around Stockholm led to an increase in the residential population in the satellites and the proportion of commuters to Stockholm (Cervero 1995). After the Second World War, the decentralization of industry, commerce, and residences was fostered by the development of roads and telecommunication systems, which provided easy access. However, network flexibility stands in contrast to network capacity. Today there is an increasing demand for high capacity networks leading to even stronger economic centers at the nodes of high capacity transportation networks (Feitelson and Salomon 2000). On the other hand, a high capacity commuter infrastructure supports the leapfrog expansion of residential settlements at the feeders, as Southworth (2001) pointed out in connection with extended motorway systems. Consequently, the distances between the places where people live and where they work have increased considerably, together with the volume of traffic in urban regions.

Motorway feeders are also favorite places for depository warehouses and shopping malls. In the last 2 decades, the latter have grown strongly, providing today not only shopping but also restaurants, theaters, or sport facilities. Subsequently, other retail and service companies are drawn to these places because potential customers are already there. These places frequently develop into new centers of interaction, so-called “edge cities,” competing with the traditional core city in accessibility and the provision of working places (Garreau 1991). Today, many urban regions do not have a clear center because different subcenters have emerged in their environs and a carpet of loosely overbuilt residential settlements has stretched out between these centers and subcenters.

Certain cities may turn into so-called “knowledge cities,” like Barcelona, Helsinki, Melbourne, or Singapore. Yigitcanlar (2009) lists the characteristics of modern knowledge cities as having a highly educated population, good provision with information and communication technology, and high quality of place. He points out that, besides the presence of universities and access to traffic and communication networks, a multicultural character, attractive public spaces, and affordable housing are indispensable strategic prerequisites to turn a city into a knowledge city.

In summary, as a consequence of the strong transitions of society, economic development in urban regions is no longer bound to places with specific natural advantages, but more depending on anthropogenic factors. However, the key driver of urbanization has always been access to the bases of production and trade. Today, accessibility itself can be a driver of urban development at a certain place, as shown by the emerging edge cities.

## ECOSYSTEM SERVICES IN URBAN REGIONS

**Figure 1:**
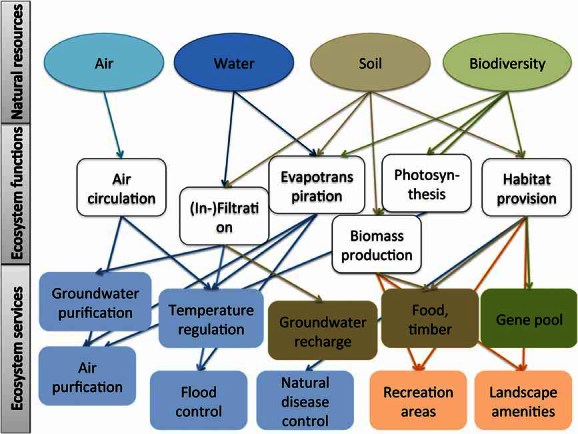
The relationships between the natural resources, ecosystem functions, and ecosystem services that are particularly important and threatened in urban regions. The ecosystem services are colored according to their categories: blue, regulating services; brown, provisioning services; orange, cultural services; green, example of supporting services.

The idea behind the concept of ecosystem service is that ecosystems provide vital goods and services for humans through the simple performance of certain ecosystem processes (Costanza et al. 1997; MEA 2005). The ecosystem processes with a potential to provide ecosystem services for humans are called “ecosystem functions,” whereas the authors do not exclude that all ecosystem processes have the potential to be ecosystem functions and provide ecosystem goods and services. Figure 1 shows the connections between ecosystem functions and services using the example of the most important ecosystem services in urban areas, like food and timber, (drinking) water, fresh air and temperature regulation, landscape amenities, and recreation areas. In the top row are the natural resources that provide the bases of these services. The next row indicates the ecosystem functions that provide the ecosystem goods and services included in the bottom rows. The colors indicate the categories of ecosystem services according to the currently widespread classification system: 1) regulating (blue), 2) provisioning (brown), 3) supporting (green), and 4) cultural services (orange) (Hanson et al. 2012; TEEB, 2010). The figure makes clear that many ecosystem functions provide more than one ecosystem good or service and, conversely, certain ecosystem services can be provided by several different and independent ecosystem functions.

The ecosystem functions depend on location specific natural factors and, consequently, the occurrence and performance of ecosystem services differ with the sites. For example, the types of natural habitat vary according to climate conditions, soil water household, and nutrient availability. In addition, some ecosystem services depend on others and, therefore, are also vulnerable to disturbances of the services they depend on, e.g., a fen with a water household disturbed by drainage loses its qualities of a natural wetland habitat. Finally, some ecosystem services are compatible with one another (e.g., a natural wetland habitat can also be used as recreation area); others exclude each other (e.g., draining a fen for crop production destroys the former habitat services). Because of these complex relationships between different ecosystem services, it may have fatal effects, if humans focus on a certain ecosystem service (e.g., the carrier service; see below) without paying attention to the natural prerequisites for other ecosystem services at the given places.

### Carrier services

With construction and transport, humans make use of solid ground, which, strictly speaking, also belongs to the goods and services of natural ecosystems. Solid ground and the corresponding carrier services can be considered one of the supporting ecosystem services. De Groot (2006) actually defined an additional class of carrier services that provide a “suitable substrate or medium for human activities and infrastructures.” Construction results in soil sealing, which particularly affects soil-borne ecosystem services like water regulation, gas diffusion, energy transfer, and biota (Salenghe and Marsan 2009). Humans have developed sophisticated construction techniques for any substratum, so that construction can take place almost anywhere, independently of the site's natural properties. This fact has enabled urban development to no longer be bound to natural conditions and puts strong pressure on all other ecosystem services.

### Cultural services

The cultural services of ecosystems are essential for human well-being, i.e., people's physical and psychological health, recreation, and place attachment. Today the demand for cultural ecosystem services is increasing and putting pressure particularly on the space for nearby recreation and landscape amenities. Although access and proximity to infrastructure (i.e., schools, shopping facilities, hospitals) are the main criteria influencing people's choice of where to live, landscape amenities become more important the higher the household income (Vogt and Marans 2004). Numerous studies demonstrate that the availability of sunny and quiet places, with open view of natural landscape features, particularly open bodies of water, or of urban parks and traditional buildings, lead to higher housing prices (Schaerer et al. 2008; Waltert and Schläpfer 2010). An increasing number of people are settling in such attractive places on the urban fringe, who want, at the same time, to be not too close to their neighbors. This is one of the causes of the unproportional recent increase in settlement area. Another is the fact that, in attractive places, the real estate market usually establishes particularly large dwelling areas with a high standard of construction, which may even be higher than what buyers actually need (Millward 2002). These developments compromise the principle of equal opportunities in 2 ways: the privatization of the landscape as a public good in particularly attractive places, and social segregation due to the market's focus on wealthy customers in these places. Increasingly, low-income households can only afford apartments in places suffering from noise and air pollution (Schaerer and Baranzini 2009).

Small and scattered settlement units are, however, beneficial for the local quality of life, because open spaces for nearby recreation are close even from the village centers. If the settlements are clearly structured so that orientation is easy, and if people can complete daily tasks, like school or shopping, on foot or by bicycle, these settlements tend to become favorite dwelling places for families with children, who typically constitute the main proportion of the population in the suburbs (Thomas and Pattaroni 2009). According to Yigitcanlar (2009), core cities will have to provide these qualities as well, if they want to become modern knowledge cities. They should provide their inhabitants with public open spaces to meet, play, and relax, particularly close to the residential areas (Kaspar and Bühler 2009). People preferably use urban parks and forests for physical exercise, or to relax and recover from stress, and usually go there on foot (Frick et al. 2007; Home et al. 2007). Although urban expansion in Europe and North America has, admittedly, been mostly accompanied by more prosperity and quality of life, it still compromises other important ecosystem services, of which people in our modern society are often not directly aware.

### Provisioning services

The provisioning services provide humans with food, water, energy, and construction material and used to be a key factor determining the location of a town. As the most fertile agricultural soils tend to be in urban regions (Nizeyimana et al. 2001), urban expansion has a particularly strong impact on food production. For the United States, Imhoff et al. (2004) estimated that the current loss of net primary production from agricultural lands through urbanization, compared to agricultural land without urbanization, equals the caloric needs of 16.5 million people or approximately 6% of the US population. Today, these losses are compensated for by food production in other regions of the world together with increased fertilizer use and irrigation. Foley et al. (2005) estimate that irrigated cropland has increased worldwide by 70% during the past 4 decades. However, much of this irrigated land has become heavily salinized, resulting in an estimated global loss of approximately 1.5 million hectares of arable land per year. Consequently, the ecological footprint of modern cities in industrialized countries on food production is far larger than the direct loss of arable land through building activities. Global climate change may even aggravate this problem, as the climate conditions in the temperate zones may become more favorable than they are today for crop production, whereas in the rest of the world they may deteriorate greatly. Parry et al. (2004) used climate change scenarios from the Special Report on Emissions Scenarios from the Intergovernmental Panel on Climate Change to predict a potential increase in crop yield of 5% to 10% for the industrialized countries up to the 2080s, whereas the developing countries will suffer from yield reductions up to 30%. If building on the most fertile soils in Europe and North America continues in the same way as today, it will become far less possible to reach this estimated increase in crop yield even under more favorable climatic conditions.

Cities are also expanding their ecological footprint toward their hinterlands in the context of groundwater consumption. Not only does intensive use of groundwater with growing population lower the groundwater table, but a city also produces wastewater that equals 75% to 100% of the total water inflow (Kennedy et al. 2007). Unpurified wastewater contaminates the natural groundwater. Consequently, many cities rely on aquifers in the urban hinterlands for their water consumption. Additional wastewater makes the groundwater table rise again, sometimes even higher than it was originally. These changes in the groundwater table may affect the ground's carrier service and cause damage to buildings (Hayashi et al. 2008).

### Regulating services

Regulating services maintain essential processes and life supporting systems. They include, among others, climate regulation, the maintenance of air quality, and the regulation of water flows. Urbanization has strong impacts on the regional water regime. Soil sealing inhibits water infiltration and causes surface run-off, leading to increased flooding in urban open waters and overstrained water purification plants. The remaining infiltration paths are greatly reduced compared to the natural situation, because they follow the underground structures of basements and underground garages to the groundwater aquifer (Nakayama et al. 2007). Consequently, the soil's water filtration capacity is reduced and open waters and groundwater may become contaminated.

Another impact on the regulating services in large cities are heat islands. The concentration of high buildings hampers air circulation and the predominance of heat-absorbing concrete, together with the absence of evaporating vegetation, lead to overheating. In addition, the high amount of energy consumed in cities is generally converted into heat. Consequently, temperature in city centers may be 7° C to 10° C higher than in the surrounding countryside (Bolund and Hunhammer 1999; Kennedy et al. 2007). The diurnal temperature range is reduced, particularly because of increased minimum temperatures (Kalnay and Cai 2003). Scattered suburban settlement patterns may, however, mitigate the heat island effect provided that the single villages do not grow together to form another city or merge with the central city.

### Supporting services

Supporting services maintain other ecosystem services. They include natural habitats and the services of their plant and animal communities (e.g., photosynthesis and decomposition of organic waste). Urbanization changes the conditions of natural habitats and has thus an impact on biodiversity. Settlements and infrastructure are barriers to the movement of many animal species, and prevent populations from mixing up. Kuehn et al. (2007) discovered stronger genetic differences between roe deer populations on different sides of a motorway in Switzerland than expected if only the geographic distance between the populations were taken into account. Landscape fragmentation has dramatically increased in Central Europe. Jaeger et al. (2007) found a decline in the mesh size of connected areas of 43% on average from 1930 to 2004 in the German state of Baden-Württemberg. Not surprisingly, the smallest patches were found in the urban areas. The reduction in patch size has an even stronger impact on species survival than the spatial separation of habitats because the smaller the patches, the larger the relative edge area with external disturbances (Di Giulio et al. 2009). In urban areas, not only are the habitat patches small but the disturbances are also strong (e.g., traffic noise, domestic animals as predators, and human recreational activities). Hence, urban regions tend to host only species that can either tolerate disturbances by humans or survive in very small habitats. Therefore, the majority of species found in cities are ubiquist species (Jules and Shahani 2003; Rickman and Connor 2003). On the other hand, topographic and environmental conditions in urban regions are usually very heterogeneous and provide habitat niches for some specialized species (e.g., species threatened by intensive agriculture) (Sattler, Duelli et al. 2010). In addition, the elevated temperatures in cities favor thermophile species (Germann et al. 2008; Nobis et al. 2009). The question today, however, is whether the species compositions in cities and conurbations are natural biocoenoses that are able to render other ecosystem services like pollination or natural disease control. Sattler, Borcard et al. (2010) have strong doubts considering spider, bee, and bird communities in Swiss cities. The spatially limited high heterogeneity of small habitat patches and the frequent disturbances have a negative impact on the equilibrium of the communities.

## CHALLENGES FOR THE PLANNING OF URBAN REGIONS

Although in the past, provisioning services used to be most important for the urban population, today, people search for cultural services rendering high quality of life in urban regions. This change of importance is mirrored, among others, in the high real estate prices at attractive residential sites. Consequently, ecosystem goods and services consumed in urban regions are increasingly provided in the hinterlands and even to some extent in other parts of the globe. This leads, in some cases, to a marked global imbalance between the regions producing ecosystem services and the regions consuming them in the long run. Other hand ecosystem services, like fresh air and a comfortable temperature regime, cannot be produced far away from their consumers. Hence the question arises of how to organize urban regions so that they are able to be as self sufficient as possible in providing the necessary ecosystem goods and services there.

As others have shown before, and as Tratalos et al. (2007) confirmed, the performance of most ecosystem services declines with increasing sealed area. High building density entails intensive soil sealing, whereas loose overbuilding provides higher landscape heterogeneity, which is favorable for both humans and nature. It offers a variety of habitat for different species and, at the same time, a more varied landscape scenery (Di Giulio et al. 2009; Home et al. 2009). However, underground soil sealing may exceed the visible surface sealing of a loose housing pattern, what hampers the soil's filtration and water purification capacity. In addition, private gardens are not accessible for the public, and thus reduce the public recreation area, as well as the area for agricultural production. These drawbacks can be avoided by combining a dense construction pattern together with the provision of, where possible unsealed, open spaces with public access. Open spaces with natural vegetation or open water bodies also mitigate the heat island effect.

The current patterns of many European city–regions with central cities and a fringe of increasingly scattered suburban villages seems to satisfy the very diverse requirements for the living space of the different groups of inhabitants best. People's wishes and needs for a residential environment vary widely, and appear to range between preferring the density of interaction in cities and the openness of the countryside in the suburbs, according to people's individual preferences and current life phase (Grêt-Regamey et al. 2012). Adolescent people frequently live in cities and benefit from the high density of public transport, cultural, and educational facilities. Young families typically settle down in the suburbs as a result of both the affordable housing prices and the ideal sizes and structures of the residential areas. Elderly people often move back to urban centers to get closer to public transport and medical supply.

Hence, what is necessary is careful planning the complex systems of urban regions rather than a tough fight against urban sprawl. This is actually a great challenge because most urban systems expand over several political entities (Steudler et al. 2004). There is a need for strict external limits on development, a move away from extensive accessibility, and concentrated development on a few focal points. The municipalities should, in addition, be encouraged to reconvert sealed soils (e.g., when reallocating derelict industrial sites and closing the gaps between buildings). This would help to reduce the net amount of sealed soil and mitigate landscape fragmentation (Schwick et al. 2012).

## BEST PRACTICE EXAMPLES FROM SWITZERLAND

The following best practice examples emphasize the 3 points where action is needed: 1) setting outer limits on settlements (sectorial plan of crop rotation areas), 2) intermunicipal cooperation on the planning of settlements and infrastructure (conurbation projects), and 3) compensation for newly sealed soil (breaking up a country road).

### Federal sectorial plan for crop rotation areas

This example has been selected because it is a planning instrument that has the purpose of preserving a specific ecosystem service (the provisioning service) and, therefore, allows for the natural factors of the location. In 1992, the federal government determined a minimum of approximately 440 000 ha of arable land (so-called crop rotation areas [CRA]) in Switzerland to be preserved from being built over. It enacted the sectorial plan for CRAs that prescribes the quantity of crop rotation areas each canton has to preserve. Each canton determines the specific locations of the CRAs in the cantonal master plan according to the criteria for assigning CRAs specified in the sectorial plan. Crop rotation areas are supposed to be those areas best suited for agricultural production.

The basic idea for this sectorial plan emerged during the Cold War to ensure the Swiss population had enough food for several months in times of crises and import interruptions. These objectives are actually obsolete today. Therefore, the federal offices of spatial planning, agriculture and environment had evaluated the sectorial plan and particularly the criteria to assign CRAs in 2003. The evaluation revealed that the cantonal planning offices value the sectorial plan very favorably because it is the only restrictive planning instrument to limit the expansion of settlements and infrastructure on agricultural land (ARE 2003). The National Forest Act dictates the preservation of the forest area and the Nature Protection Act ensures areas of nature protection are saved from being built over, but there is no law prescribing the protection of agricultural land.

I was the leader of the project to evaluate and update the criteria to assign CRAs, which was accomplished by the Swiss Soil Science Society. We mainly confirmed and specified the original criteria because they proved to be useful and considerable changes to the criteria might have threatened the legitimation of some existing CRAs. However, the update of the CRA criteria was not meant for the evaluation of existing CRAs.

The criteria describe the soil's ability of food and fodder production, which depends on climatic conditions, soil depth, and properties. The climate criterion includes the regions with the most favorable conditions for crops and grassland in every canton. The key criterion on soil quality is the soil depth, which the crops can use as rooting zone. This must be at least 50 cm. It basically represents the sum of the topsoil and subsoil horizons and includes several subtractions according to the range of groundwater table differences, impervious horizons, and the coarse fraction. In cantons without area-wide soil maps, rootable soil depth was ascertained with a minimum of auger holes at specific sites and the final extent of the CRAs that entered the cantonal master plans was determined due to the topography's suitability for management with farming machinery (i.e., inclination of the soil surface being <18%). Our group confirmed this criterion because flat areas are most threatened by construction. We finally suggested 2 additional criteria considering soil contamination and compaction for assigning new CRAs, particularly in urban environs and on restored soils.

From an environmental perspective, it does actually not suffice to preserve only the most productive soils from construction. The sectorial plan should be adjusted to promote the goals of sustainable development and extended to preserve all soil-borne ecosystem services. However, the soil's food production capability depends on several additional soil functions, what is particularly accounted for with the composite parameter of rootable soil depth. Its ascertainment requires the estimation of the soil's air, water, and temperature household and nutrient availability. Consequently, it encompasses not only information about the provisioning soil services of food and fodder production, but also about the regulating services contributing to the soil water household and the supporting services of providing natural habitat for vegetation. For this reason, our group refrained from suggesting more detailed parameters describing soil water household or natural habitat quality.

Practice shows that urban cantons with strong population growth, in particular, find it difficult to maintain the prescribed quantity of CRAs or have already fallen short. As a consequence, cantonal planning offices have started to discuss ways of reducing the mandatory number of CRAs with the federal office or of compensating for CRAs on highly productive soils in urban areas that have been built over by designating new CRAs beyond the urban fringes. In most cases, these new CRAs would have to be areas on less productive soils than the built over CRAs. Thus, the cantons are considering balancing this by having larger areas classified as CRA than before.

### Conurbation projects

The conurbation projects are an innovative approach for comprehensive planning. In 2001 the federal government launched a new policy for conurbations to support cities and the municipalities in solving their urgent problems in the urban fringes, particularly to mitigate some burdens the core cities have, such as traffic congestion (http://www.are.admin.ch/themen/agglomeration). The policy is intended to intensify intermunicipal cooperation and support joint projects to solve common problems with financial incentives. In such a project, the local authorities in the core city, the suburban municipalities, and the canton would jointly set up a program to coordinate and organize the development of the entire urban region. In Switzerland, the municipalities are autonomous in developing their zoning plans, which is why voluntary cooperation would probably be most effective. Such projects should encourage the municipal councils to think in terms of the whole urban areas and to coordinate planning and development, although the different political entities have different planning and construction legislation. This is particularly challenging in conurbations expanding over several cantons, like the city–region of Zurich, or across national boarders into neighboring countries, as in the regions of Geneva or Basel.

In reality, most conurbation projects have concentrated on improving the local traffic systems mainly because the most urgent problems they face are traffic congestions and overstretched public transport systems, but also because an infrastructure fund was established in 2008 that provides money for additional transport infrastructure. The recently completed evaluation report on the first 10 years of this federal policy indicates that intermunicipal cooperation has worked well but criticizes the narrow focus on traffic problems (ARE, SECO 2011). Therefore, future conurbation projects will place more importance on planning settlements and open spaces cooperatively. The Federal Office of Spatial Planning published a guide on concentrated development (ARE 2009), to which I contributed as an expert for landscape and environment. The guide does not mention ecosystem services directly, but it emphasizes the open spaces within and outside the overbuilt areas. The guide recommends beginning with the development planning by analyzing the open spaces with unsealed surfaces. This encompasses their location and extension and, in particular, their importance for aesthetic views and nearby recreation (cultural services), natural habitat (supporting services), and temperature regulation (regulating services). It further stresses the connectivity of these open spaces to be indispensable for fulfilling the functions mentioned. The measures to take include preserving greenbelts between the different settlement units, upgrading the environs of open waters as seminatural habitats and recreation areas, and creating public parks at places where connectivity is interrupted. First attempts are made in the conurbation projects of the city–regions of Zurich and Geneva.

This refocusing is necessary to ensure the conurbation projects really help maintain ecosystem services in city–regions. As the cantons and municipalities are responsible for spatial planning, the only way the federal government can encourage comprehensive settlement planning at a regional level is to provide incentives for voluntary initiatives. Raising awareness among the municipal councils about the benefits of ecosystem services and how essential they are would certainly support the original idea of the conurbation projects.

### Breaking up a country road

**Figure 2:**
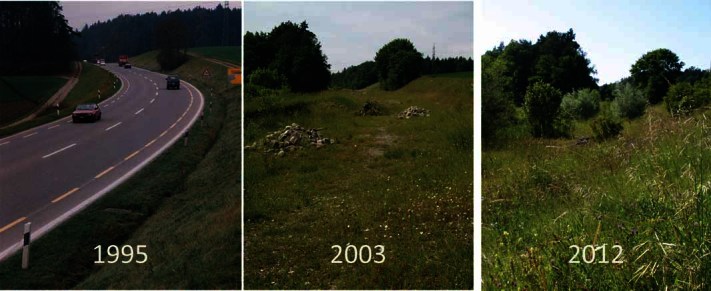
In 1996, 2 km of this country road, ca. 30 km north of the city of Zurich (left picture), was dismantled as an ecological compensation measure for the construction of a motorway on neighboring agricultural land. The pictures to the right show the vegetation's development on the gravel parent material 7 and 16 years after restoration (47° 33.4′ N; 8° 42.2′ E). Photographs by M. Fries (1995) and S. Tobias (2003 and 2012).

This is one of the very few examples of sealed soil being uncovered and then left open. A country road connecting 2 villages in the greater urban region of Zurich became obsolete for transit traffic when a nearby motorway opened in 1996. The motorway can carry much more traffic than the former country road and serves as a bypass road relieving the former transit villages, which suffered from noise, air pollution, and traffic congestion. The bypass required sealing of a 4 km stretch of agricultural land. In return, 2 km of the old country road were dismantled between the 2 villages as ecological compensation (Figure 2). Ecological compensation measures are compulsory for large construction projects to build roads, railway lines and other infrastructure and should consume something in the range of 3% of the total construction costs. In this case, the compensation measure could be accomplished on land owned by the canton and no negotiations with private landowners were necessary.

I led a students' thesis in 2000 investigating this example because it is interesting for 2 reasons. First, the shortest connection between the 2 villages is now only passable with human-powered mobility or agricultural vehicles. Second, this is a pioneer example of a way to combat landscape fragmentation. A smaller forest that was isolated due to the country road and its high traffic volume is now reconnected to the main forest. A small river that used to cross the country road and the adjacent agricultural land in an underground pipeline was opened, and its shorelines were replanted with endemic shrubs and bushes. This river now connects the newly created habitats for pioneer species on the old road's bottom line to a nearby wetland. Finally, new recreation areas have been established and the quality of life in the villages has greatly improved as a result of the project.

The project created notable benefit in respect of cultural ecosystem services, whereas the loser of the game was agriculture that makes use of the provisioning services. According to the soil map, the soils lost to the new motorway were deep and fertile sandy loams with high air infiltration and water storage capacities. Together with a soil-mapping expert, I excavated 2 soil profiles in the renaturalized part of the old country road in the year 2012 (i.e., 16 years after the reconversion). We could only dig 25 cm deep and reached an extremely dense horizon of sandy loam that had artificially been compacted to serve as a stable ground for the country road. This water-impervious layer is the reason for several ponds. A proper topsoil layer (A-horizon) only occurred in the top 1.5 to 5 cm immediately followed by the parent material (C-horizon) without any weathering horizon (B-horizon). The thicker topsoil was found at a site with 35% to 45% coarse material all over the soil profile. The high amount of the coarse fraction supported air infiltration and thus biological activity. The rate of natural soil formation (regulating service) has been something between 1 and 3 mm per year, and future soil development mainly depends on how thick the compacted layer is and how fast roots and microbes can loosen it. It is not clear which ecosystem services will develop in the future, if the soil will ever be suitable for crop growth again, if specialized wetland ecosystems with fens can build up, or if mediocre habitats for ubiquist species will develop.

## DISCUSSION AND CONCLUSION

Today ecosystem services are strongly threatened in urban regions, because economic production is independent of location-specific natural factors and, consequently, the people have become less aware of the importance of ecosystem services. In addition, accessibility has excessively spread and urban regions provide almost no more places without any disturbances. The literature reviewed does not indicate an optimum settlement pattern that supports all ecosystem services in urban regions equally. Many urban regions in Europe have both a high building density in the central city and in the immediately neighboring municipalities and increasingly scattered settlements toward the edges of the urban fringe. As this review has shown, this settlement pattern can offer a high diversity of ecosystem services, as long as the single settlement units have clear external limits and offer attractive public green spaces.

Spatial planning can contribute to the preservation of ecosystem services in 3 ways: 1) setting outer limits on settlements, 2) encouraging intermunicipal cooperation on comprehensive planning with single focal points of development, and 3) compensating for new soil sealing.

The Swiss examples presented show ways to carry out these tasks. The sectorial plan of crop rotation areas emphasizes the provisioning services and intends to counteract the ongoing outsourcing of food production to faraway regions. The conurbation projects aim at preserving the location specific natural features enhancing quality of life. Finally, the reconversion of sealed soil is an indispensable measure to reestablish any lost ecosystem services. However, planners face serious difficulties when implementing these approaches. With continuously growing urban regions, the cantons will probably increase the pressure on the federal spatial planning office to reduce their mandatory quantity of CRAs or to let them compensate for CRAs with larger areas on less productive soils. This would run counter the goals of the sectorial plan and future generations may not be able to cultivate the same crops as today. The conurbation projects' principle of incentives and voluntary cooperation forces the municipalities to declare their willingness to cooperate before starting a project. This certainly makes the project's implementation easier, but, nevertheless, a conurbation project still requires hard negotiation about the focal points of development and, in particular, about abdication of development and accessibility—what the municipalities affected will not easily accept. Finally, breaking up sealed soil can fail because of the property rights on the land considered. Most private landowners will not agree to leave their land open, because they fear a loss of its monetary value. Even if the property rights are opportune like in the example presented, it may be questioned whether the ecosystem services that can be restored compensate for those lost to soil sealing. More research is needed on the possibilities and limits to restore ecosystem services on reconverted areas.

To conclude, the efforts of spatial planning can only work out if the responsible decision makers are willing to preserve ecosystem services in urban regions. Otherwise, the planning instruments risk annulment, as the negotiation about CRAs demonstrates. Because spatial planning is in the domain of local or regional authorities and policy makers in most countries, it is very important to educate the local decision makers about the importance of ecosystem services in urban regions.

## EDITOR'S NOTE

This paper is one of 8 articles generated from the SETAC Special Symposium: Ecosystem Services, from Policy to Practice (15-16 February 2012, Brussels, Belgium). The symposium aimed to give a broad overview of the application of the ecosystem services concept in environmental assessment and management, against the background of the implementation of the European environmental policies such as the biodiversity agenda, agricultural policy, and the water framework directive.
